# Prognostic factors for the efficacy of infliximab in patients with luminal fistulizing Crohn’s disease

**DOI:** 10.1186/s12876-023-02676-9

**Published:** 2023-03-08

**Authors:** Ye Ma, Runfeng Zhang, Wei Liu, Yinghao Sun, Jingnan Li, Hong Yang, Hong Lv, Yue Li, Bei Tan, Xiyu Sun, Jiaming Qian, Ji Li

**Affiliations:** 1 Department of Gastroenterology, Peking Union Medical College Hospital, Beijing, China; 2 Peking Union Medical College and Chinese Academy of Medical Sciences, Beijing, China; 3 Department of Radiology, Peking Union Medical College Hospital, Beijing, China; 4 Department of General Surgery, Peking Union Medical College Hospital, Beijing, China

**Keywords:** Crohn’s disease, Fistula, Infliximab, Prognostic factors, Efficacy

## Abstract

**Background:**

Enteric fistula is one of the penetrating features in Crohn’s disease (CD). This study aimed to clarify the prognostic factors for the efficacy of infliximab (IFX) treatment in luminal fistulizing CD patients.

**Methods:**

We retrospectively included 26 cases diagnosed with luminal fistulizing CD hospitalized in our medical center from 2013 to 2021. The primary outcome of our research was defined as death from all causes and undergoing of any relevant abdominal surgery. Kaplan–Meier survival curves were used to describe overall survival. Univariate and multivariate analyses were used to identify prognostic factors. A predictive model was constructed using Cox proportional hazard model.

**Results:**

The median follow-up time was 17.5 months (range 6–124 months). The 1- and 2-year surgery-free survival rates were 68.1% and 63.2%, respectively. In the univariate analysis, the efficacy of IFX treatment at 6 months after initiation (*P* < 0.001, HR 0.23, 95% CI 0.01–0.72) and the existence of complex fistula (*P* = 0.047, HR 4.11, 95% CI 1.01–16.71) was found significantly related to the overall surgery-free survival, while disease activity at baseline (*P* = 0.099) also showed predictive potential. The multivariate analysis showed that efficacy at 6 months (*P* = 0.010) was an independent prognostic factor. The C-index of the model for surgery-free survival was 0.923 (*P* < 0.001), indicating an acceptable predictive effect.

**Conclusion:**

Prognostic model including the existence of complex fistula, disease activity at baseline and efficacy of IFX at 6 months may be useful to predict long-term outcome of luminal fistulizing CD patients.

## Introduction

Crohn’s disease (CD) is a chronic, recurrent inflammatory bowel disease characterized by transmural inflammation affecting the entire digestive tract and especially the distal ileum. Multiple factors including intestinal immune dysfunction, intestinal mucosal barrier destruction, intestinal microbiota abnormalities, and genetic and environmental factors are believed to contribute to the pathogenesis of CD [1, 2]. Fistula is common due to the transmural inflammation of CD, and the proportion of luminal fistulizing CD reaches 5–10% [3]. It has been reported that the overall surgical rate approaches 50% [4], while the clinical recurrence rate approaches 25% at 2 years after surgery [5].

The anti-tumor necrosis factor-α (TNF-α) monoclonal antibody represented by infliximab (IFX) has been widely used in the treatment of CD in the past 2 decades. Several studies have confirmed that IFX is a relatively fast and safe choice that can effectively improve clinical symptoms, inhibit inflammatory reactions, promote mucosal healing and closure of the perianal fistula [6], improve patient quality of life [7], achieve clinical and endoscopic remission, and reduce surgical rates [8]. However, IFX is not effective in 20–40% patients due to loss-of-response [9]. Furthermore, IFX might lead to increased risk of infection and high economic costs. Although a meta-analysis in 2018 showed that IFX treatment for fistulizing CD improved prognosis with a cumulative RR of 2.01 (95% CI 1.36–2.97) [10], the efficacy and suboptimal response of IFX therapy in luminal fistulizing CD is still challenging due to the limited number of published studies.

The incidence of CD in China is increasing yearly [11]. A multicenter study based on Chinese CD patients showed that 57% of patients were at risk of experiencing a suboptimal response to first-line anti-TNF therapy 2 years after its initiation in China [12].

There is no current prediction model for the response to IFX treatment for luminal fistulizing CD (lfCD) patients. This study aims to provide a detailed description of the long-term prognostic markers of lfCD patients and to further explore the prognostic factors associated with IFX treatment and provide more rational and individuallized therapy for these patients.

## Patients and methods

### Patients

A comprehensive search of medical records was performed to identify lfCD patients diagnosed and hospitalized at our medical center between January 2013 and June 2021. Patients meeting all of the following inclusion criteria simultaneously were included: patients with diagnosed CD hospitalized at our medical center, intestinal fistula confirmed by computerized tomography (CT), magnetic resonance imaging (MRI), ultrasonography or endoscopy; follow-up ≥ 6 months; and ≥ 3 courses of IFX treatment. Patients meeting any of the following exclusion criteria were excluded: surgical-related fistula; follow-up < 6 months; < 3 courses of IFX treatment due to any reason; and previous exposure of IFX before diagnosis. Twenty-six patients were finally included in the study.

### Data collection

Baseline clinical profiles were collected and reviewed, including sex, age, Montreal classification [13] and baseline treatment. The detailed baseline clinical characteristics of CD were evaluated at the time of diagnosis of intestinal fistula. The Crohn’s Disease Activity Index (CDAI) was used to evaluate the disease activity in brief [14]. The characteristics of the fistula were classified by type, location, and the existence of a complex fistula. The fistula was defined as complex fistula if there were multiple fistula tracts or the patient has history of abdominal abscess. History of abdominal infection or abcess were also collected.

Laboratory data related to CD were also collected, including blood routine, albumin, erythrocyte sedimentation rate (ESR), and C-reactive protein (CRP). Due to the non-availability of data, serum IL-6 and TNF-α level and other indicators were not collected and included in the analysis. Serum IFX concentration were also not included.

Imaging data (enhanced abdominal and pelvic CT, CT enterography, or MR enterography) at baseline were reappraised. Data were collected including the characteritics of fistula, and other signs of CD activity, including thickening or strengthening of the intestinal wall, existence of stricture, thickened mesenteric vessels and enlarged mesenteric lymph nodes.

### Follow-up and evaluation

Treatment and follow-up profiles were partially retrieved from the medical records. All patients were also contacted via telephone by researchers in November 2021 to obtain updated follow-up information, including details of medical regimens, survival status and acceptance of surgical operation. Therapeutic regimens included IFX, glucocorticoids, anti-inflammatory agents, immunosuppressants, and probiotics. The efficacy of IFX treatment was determined based on improvements in the CDAI score (remission, CDAI < 150; response, CDAI ≥ 150 points and decreased by ≥ 70 points; non-response, CDAI ≥ 150 points and decreased by < 70 points).

The primary outcome of our research was defined as death from all causes, and undergoing of any abdominal surgery related to fistula. The secondary outcome was discontinuation of IFX treatment for any reason.

### Statistical analysis

Data analysis was performed with SPSS version 25.0 (SPSS, Inc., Chicago, IL, USA). Continuous variables are shown as the median and interquartile range. Categorical data were summarized as the percentage of the whole research population. Continuous variables were transformed into categorical variables, mainly based on routine cutoff values used in clinical application, for example, 35 g/l for serum albumin and 18.5 kg/m^2^ for body mass index (BMI). Overall survival was calculated from the beginning of treatment until the primary outcome. Survival curves were plotted with the Kaplan–Meier method. The log-rank test was employed to screen for variables that might be associated with survival. Only variables with certain significance (*P* < 0.10) in the univariate analysis were considered to be possibly associated with survival and further included in the multivariate analysis. The Cox proportional hazard model was used to assess the association of variables with survival. Hazard ratio (HR) and 95% confidence interval (CI) was used to represent the results of analyses [15, 16].

## Results

### Baseline clinical characteristics of CD patients with luminal fistula

The baseline clinical characteristics of CD patients with intestinal fistula are shown in Table 1. Among all the 26 patients, 21 of them were male (80.8%). The median age at the time of diagnosis is 27.5 years (range, 17–64 years). Six cases had a histroy of cigrette consumption, 1 of them was smokers and 5 were former smokers. Five cases had MRI as part of the evaluation. Twelve cases had theraputic drug monitoring and dose adjustment (if necessary). Thirteen cases were diagnosed with complex fistula. Thirteen cases had a history of abdominal infection before IFX initiation, while 6 cases had a history of abdominal abscess. According to the CDAI score, 4 cases were in clinical remission stage (CDAI < 150 points), 9 cases were in a mild active stage (150 ≤ CDAI < 220 points), and 13 cases were in moderate-to-severe active stage (CDAI ≥ 220 points) at baseline. Thirteen cases received total elemental enteral nutrition, 24 cases received glucocorticoids, 23 cases received 5-aminosalicylic acids, and 13 cases received immunosuppressants at initiation of IFX treatment.

**Table 1:** Clinical characteristics of luminal fistulizing CD patients at baseline

Clinical characteristics	N, % (Total N = 26)
Demographic features	
Age, year	27.5 (24–34)
Male	21, 80.8%
Cigrette consumption	6, 23.1%
Present smoker	1, 3.8%
Former smoker	5, 19.3%
Clinical features	
Montreal classification*	
A classification	
A1	7, 26.9%
A2	16, 61.5%
A3	3, 11.5%
L classification	
L1	6, 23.1%
L3	20, 76.9%
B classification	
B3	10, 38.5%
B2 + 3	16, 61.5%
P classification	
O	12, 46.2%
P	14, 53.8%
Features of fistula	
History of abdominal infection	13, 50.0%
History of abdominal abscess	6, 23.1%
Complex fistula**	13, 50.0%
Multiple fistula tract	11, 42.3%
Type of fistula	
Enteric fistula	19, 73.1%
Enterovesical fistula	6, 23.1%
Enterocutaneous/enteroumbilical fistula	1, 3.8%
Entero-retroperitoneal fistula	2, 7.7%
Location of fistula	
Ileum	10, 38.5%
Colon	3, 11.5%
Ileum and colon	13, 50.0%
Complicated with abdominal infection	13, 50.0%
Low body mass index (BMI < 18.5 kg/m^2^)	8, 30.8%
Decreased serum albumin (Alb < 35 g/L)	4, 15.4%
Elevated ESR***	11, 42.3%
Increased hsCRP level (hsCRP > 3 mg/L)	17, 65.4%
Baseline disease activity	
Clinical remission	4, 15.4%
Mild active	9, 34.6%
Moderate active	13, 50.0%
Severe active	0, 0
Imaging features (CT)	
Intestine stricturing	14, 53.8%
Intestine wall thickening and strengthening	16, 61.5%
Mesenteric vessel thickening	7, 26.9%
Mesenteric lymph nodes enlarging	18, 69.2%
MRI evaluation	5, 3.8%
Baseline treatment	
Total elemental enteric nutrition feeding	14, 53.8%
Glucocorticoids	24, 92.3%
5-Aminosalicylic acids	23, 88.5%
Immunosuppressants	13, 50.0%
Therapeutic drug monitoring and dose adjustment	12, 46.2%

*Montreal classification: A: age, A1: < 17 years, A2: 17–40 years, and A3: > 40 years; L: location affected, L1: ileum, L2: colon, L3: ileum and colon; B: behavior, B3: penetrating, B2 + 3 stricturing and penetrating, P: perianal lesions, O: no perianal lesion

**Complex fistula: defined as multiple fistula tract or abdominal abscess

***Elevated ESR: ESR > 20 mm/h for females, > 15 mm/h for males

### Efficacy evaluation of IFX treatment

Our research was conducted with a median follow-up time of 17.5 months (6–124 months). The results of efficacy evaluation of IFX treatment are shown in Table 2. Limited by the accessibility of clinical profiles, some patients were not evaluated at certain time points. According to the efficacy evaluation, 12 of 24 cases achieved clinical remission and 12 cases had clinical response at 3 months after IFX treatment. Fourteen of 23 cases achieved remission, 7 cases had response, and 2 cases had non-response at 6 months. Six of 8 cases achieved remission, 1 case had response, and 1 case had non-response at 24 months. The 1-year and 2-year preserve rate of IFX treatment was 60.9% (14 of 23 cases) and 50% (9 of 18 cases), respectively.

**Table 2:** Results of follow-up and efficacy evaluation of IFX treatment

Follow-up and efficacy evaluation results	N, % (Total N = 26)
Outcomes	
Death	0, 0
Surgery	8, 30.8%
1-year surgery-free survival	15 of 22, 68.1%
2-year surgery-free survival	12 of 19, 63.2%
Surgery-free survival	18, 69.2%
Fistula closure	5, 19.2%
Fistula remaining	13, 50.0%
IFX failure	3, 11.5%
Intolerance	1, 3.8%
Loss-of-response	2, 7.7%
IFX-failure-free surgery-free survival	15, 57.7%
Efficacy evaluation	
Efficacy at 3 months	
Remission	12 of 24, 50.0%
Response	12 of 24, 50.0%
Non-response	0 of 24, 0
Efficacy at 6 months	
Remission	14 of 23, 60.9%
Response	7 of 23, 30.4%
Non-response	2 of 23, 8.7%
Efficacy at 24 months	
Remission	6 of 8, 75%
Response	1 of 8, 12.5%
Non-response	1 of 8, 12.5%

There was no death case happening during the entire follow-up period. Eight cases underwent fistula-related surgical treatment, and 3 cases stopped using IFX due to intolerance (1 case) or loss of response (2 cases). Totally 18 cases reached surgery-free survival, among which 5 cases reached fistula closure and 13 cases remained survival with fistula. The rate of fistula closure was 19.2% in summary. And totally 15 cases reached IFX-failure-free surgery-free survival.

**Fig. 1:**
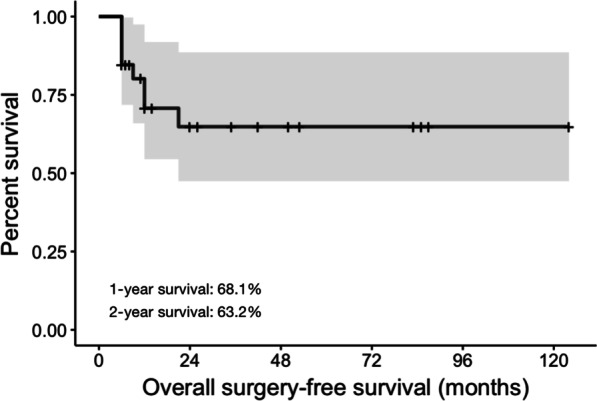
Kaplan–Meier curve of surgery-free survival of all patients with luminal fistulizing Crohn’s disease

The 1-year and 2-year rate of surgery-free survival was 68.1% (15 of 22 cases) and 63.2% (12 of 19 cases), respectively (Fig. 1). The overall median survival was not reached, and the median time from IFX treatment to surgery for patients who underwent surgery was 8 months (range, 6–25 months).

### Survival analysis

**Fig. 2:**
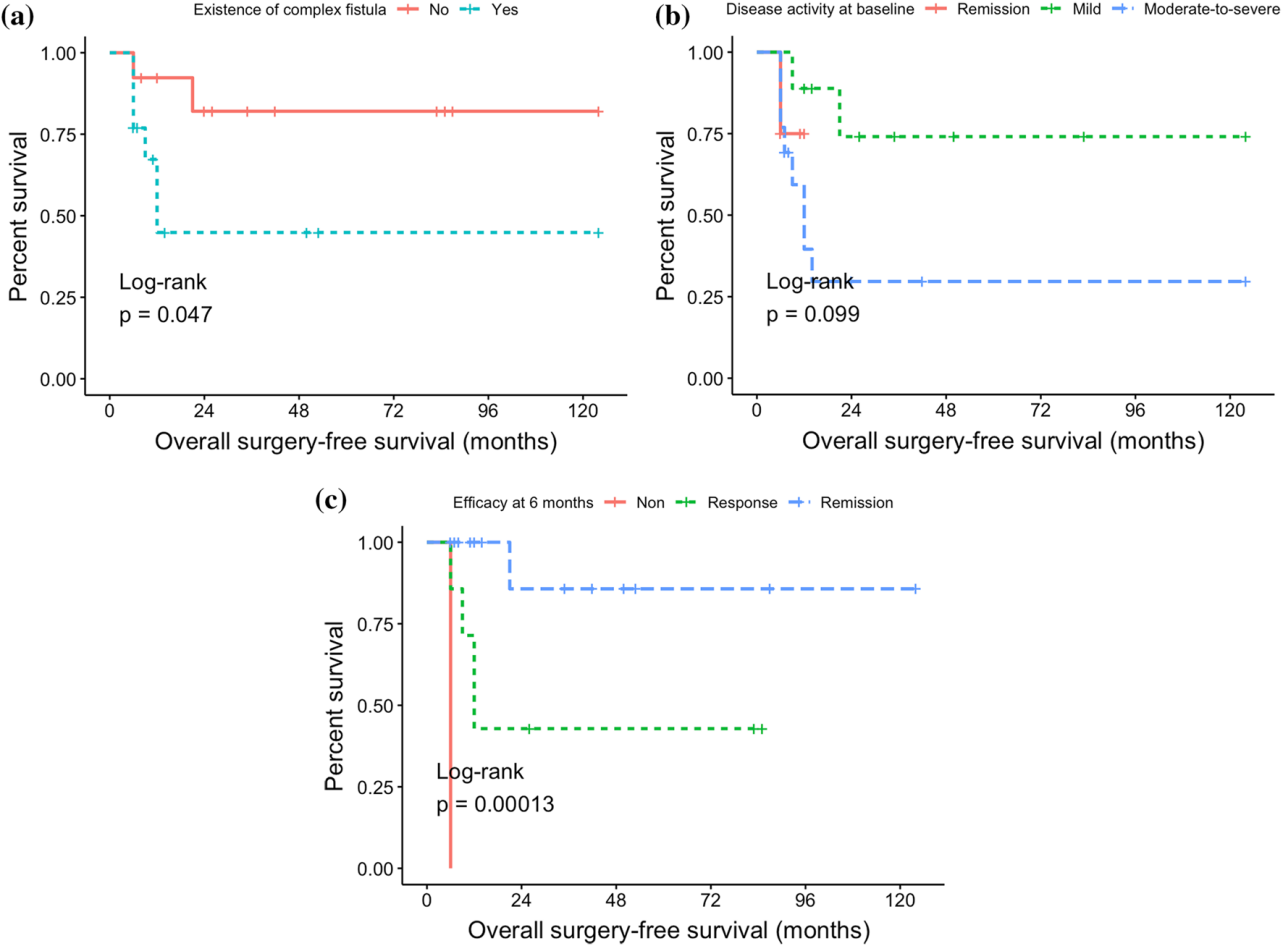
Kaplan–Meier curves according to different prognostic factors of overall surgery-free survival. **A** Existence of complex fistula, **B** disease activity at baseline, **C** efficacy of IFX at 6 months

The results of the univariate and multivariate analysis are shown in Table 3. Prognostic factors selected from the univariate analysis included: Efficacy of IFX treatment at 6 months after initiation (*P* < 0.001, remission vs. response: HR 0.23, 95% CI 0.01–0.72) and the existence of a complex fistula (*P* = 0.047, yes vs. no: HR 4.11, 95% CI 1.01–16.71) with statistical significance. Disease activity at baseline (*P* 0.099, moderate-to-severe active vs mild active: HR 2.36, 95% CI 0.77–7.19) was also selected as a variable in the multivariate analysis (Fig. 2). Other factors, including age, sex, Montreal classification, features of the fistula, baseline treatment, history of abdominal infection or abcess, decreased BMI (< 18.5 kg/m^2^), decreased serum albumin (< 35 g/L), elevated ESR, elevated hsCRP (> 3 mg/L), and imaging characteristics (including intestinal wall thickening or strengthening, intestinal stricturing, mesenteric vessel thickening and mesenteric lymph nodes enlarging) had no significant impact on overall surgery-free survival.

**Table 3:** Univariate and multivariate analyses for different prognostic factors for surgery-free survival in patients with luminal fistulizing CD

Variables	Univariate analysis			Multivariate analysis		
	HR	95% CI	*P* value	HR	95% CI	*P* value
Existence of complex fistula*	4.11	1.01–16.71	0.047**	4.32	0.85–21.93	0.104
Disease activity at baseline*	2.36	0.77–7.19	0.099	2.37	0.62–9.00	0.199
Efficacy at 6 months*	0.23	0.01–0.72	< 0.001**	0.08	0.01–0.46	0.010**
Sex	1.51	0.25–9.14	0.596	–		
Cigrette consumption	2.82	0.83–9.51	0.147	–		
Montreal classification-A	3.06	0.63–14.74	0.519	–		
Montreal classification-L	2.07	0.40–10.78	0.469	–		
Montreal classification-B	1.70	0.40–7.19	0.493	–		
Montreal classification-p	0.46	0.11–1.85	0.250	–		
Location of fistula	4.47	0.34–58.09	0.218	–		
History of abdominal infection	1.26	0.31–5.07	0.733	–		
History of abdominal abcess	3.48	0.10–125.14	0.166	–		
Low BMI	1.62	0.38–6.93	0.531	–		
Decreased serum albumin	0.47	0.11–4.87	0.770	–		
Increased hsCRP	1.26	0.28–5.76	0.765	–		
Elevated ESR	0.34	0.09–1.37	0.147	–		
Intestine stricturing	0.59	0.13–2.61	0.456	–		
Intestine wall thickening and strengthening	0.60	0.12–2.96	0.469	–		
Mesenteric vessel thickening	1.15	0.21–6.28	0.856	–		
Mesenteric lymph nodes enlarging	0.97	0.19–5.06	0.972	–		
Theraputic drug monitoring and dose adjustment	0.39	0.11–1.36	0.202	–		

*These three variables were included in the multivariate model

**P < 0.05 is considered statistically significant

The above three factors were included in the Cox proportional hazards model and a multivariate regression analysis was performed. Efficacy at 6 months was the only independent factor associated with overall surgery-free survival (*P* = 0.010). The C-index of the model for surgery-free survival was 0.923 (*P* < 0.001), indicating an acceptable predictive effect.

## Discussion

The surgery rates in luminal fistulizing CD patients are usually of concern to physicians, especially when IFX treatment. And the factors that could predict the prognosis in each patient individually are also to be considered. These factors should be evaluated with priority in clinical practice, especially in developing countries with limited medical resources, considering the benefit-harm balance and the cost-efficacy balance of IFX. Given the rarity and the complicated etiology and limited treatment choice, the majority of previous studies evaluating the response to IFX therapy in CD patients did not specifically focus on the subgroup of luminal fistulizing CD [17]. It is challenging to identify a variable consistently demonstrating to be a predictor and to establish a prognostic model of the response to IFX and rate of surgery for these patients [18]. Based on our single-center experience, we found that the 1-year and 2-year surgery-free survival rate of luminal fistulizing CD patients was 68.1% and 63.2%, respectively, and established a Cox proportional hazard model to predict the surgery rate, which included three baseline and dynamic clinical variables (existence of complex fistula, disease activity at baseline and efficacy of IFX treatment at 6 months).

The prognostic factors included in our model, derived from dozens of parameters through statistical analysis, have unique clinical significance. Unsurprisingly, the efficacy of IFX treatment at 6 months was an independent significant prognostic factor for surgery-free survival of patients. Actually IFX therapy includes an induction period of the first 3 months and a subsequent maintenance period in clinical practice. Laboratory and imaging examinations should be actively conducted for patients to evaluate their response to IFX within 3–6 months after initiation, and patients with poor response should initiate replacement of IFX with other medications and even surgery. It is reasonable that patients might continue IFX treatment and do not need to undergo surgery if clinical response is maintained after the induction period. We noted that, compared to efficacy at 6 months, efficacy of IFX at 3 months did not show a predictive significance. Secondary loss-of-response, mostly due to the anti-IFX antibody that may occur within 3–6 months or longer after IFX initiation influences the rate of IFX preservation [19, 20], which makes efficacy at 6 months a better predictive factor.

The baseline disease activity and the existence of complex fistula were also included in the model. First, as expected, disease activity at baseline contributed to surgery-free survival of patients. This is consistent with the clinical impression that more severe disease can influence the patient's ability to tolerate anti-TNF-α therapy and increase the tendency of physicians to perform surgical intervention [18]. Second, the existence of a complex fistula also contributed to overall survival. Complex fistula tend to cause more severe clinical symptoms, which also increase the tendency for surgery. A multicenter retrospective cohort study in France in 2020 reported that the existence of complex fistula was associated with the rate of surgical intervention [21], which is consistent with our findings.

We also expected that low BMI (BMI < 18.5 kg/m^2^) might contribute to a poor prognosis. First, low BMI means poor general condition, poor nutritional status, and heavy disease burden and depletion, making it more difficult for the patient to tolerate IFX treatment. At the same time, given impact of the BMI in the evaluation of CDAI, lower BMI was associated with a more serious judgment made by physician, increasing the possibility of surgical intervention [22]. However, we did not find a significant association of low BMI with poor prognosis, which might due to the limited sample size.

According to the findings of the retrospective GETAID trail, the existence of complex fistula, intestinal stricture, and elevated CRP level were associated with the rate of surgical intervention [21]. The study also reported the rate of fistula healing after IFX therapy and suggested that fistula persistence was closely related to the surgical decision. Consistently, according to our research, the existence of complex fistula was associated with the rate of surgical intervention. However, our research showed that a significant number of patients remained survival with fistula after IFX treatment and did not undergo surgery. Considering that the evaluation of IFX treatment includes both clinical response and fistula healing, the improvement of the patient's clinical symptoms may contribute to the continuation of IFX treatment despite the persistence of fistula.

From the view of cost-effectiveness, a top-down approach based on an initial induction regimen with IFX and immunosuppressants followed by IFX or glucocorticoids proved to be dominant to step-up approach with initial glucocorticoid therapy [23]. It is also reasonable to assume that initial treatment of fistulizing CD and baseline treatment at IFX initiation are associated with a prognosis [24]. Given that the patients included in our research were from the same center, the baseline treatment was similar and no positive results were found. Confounding factors could be that patients with baseline therapy including immunosuppressants tend to be in a more severe state of disease, which increases the risk of undergoing surgical intervention and masking the effect on prognosis.

Furthermore, we believe that alternative therapy is also a key factor that affects IFX compliance. In the American Gastroenterological Association Institute technical review of moderate to severe CD, adalimumab, vedolizumab, and ustekinumab are mentioned as an optional substitution for IFX [25], and have been available to patients in Europe and the USA for several years. A meta-analysis showed that they are comparable to IFX in terms of inducing or maintaining remission in fistulizing CD [10]. Before 2021, these alternative treatments were difficult to obtain or a huge financial burden because such biological agents are not marketed or insured in China. As a result, some patients can only undergo surgery when IFX treatment fails, which might influence the outcome.

Our findings further validated the efficacy of IFX in lfCD patients, a population that has usually been ignored in previous studies. Our research confirmed the effect of IFX on improving non-operative survival in lfCD patients, which is consistent with current findings in CD patients [26]. However, we also focused on the issue of IFX-failure as the secondary outcome, reporting 3 patients with IFX-failure due to intolerance or loss-of-response during the follow-up period. Regretfully, due to the small sample size, it is difficult to analyze the factors related to IFX-failure. A long-term study in CD patients receiving IFX treatment identified several predictors of IFX-failure free survival, including prior anti-TNF therapy, no drug monitoring, and early dose optimisation [27]. However, different subgroups of CD patients may have different characteristics, and patients with lfCD tend to be with higher disease activity, which increases the risk of IFX-failure, leading to inconsistent conclusions of our study.

Current researches on fistulizing CD focus more on perianal fistulizing CD (pfCD). A systematic review on pfCD patients suggests that there is insufficient evidence to conclusively suggest a predictive prognostic factor for pfCD [28]. However, there is evidence that prolonged IFX therapy in patients with pfCD may increase the benefit [29]. Additional evidence suggests that dose monitoring and higher serum concentrations are associated with better clinical outcomes [27, 30, 31]. Similarly, drug concentration monitoring and dosing optimization could be expected to be equally beneficial for the outcome of patients with lfCD, but in our study, concentration monitoring did not improve nonsurgical survival, possibly due to the tendency of multidisciplinary teams to promote surgical procedures in clinical decision-making for patients with suboptimal drug concentrations, leading to inconsistent conclusions. In addition, patients who initially respond well to IFX may not receive further monitoring subsequently, which may also influence our conclusions.

Abdominal MRI is also an important reference in the diagnostic evaluation of CD patients [32]. However, due to cost-effectiveness factors, MRI has not been applied to all patients in our center.

Our research had some limitations. First, due to its retrospective design, the baseline assessment was not totally standardized. For example, laboratory tests at different time may have different reference values, and the criteria of endoscopic evaluation may also change after time. Second, limited by the sample size, we could not eliminate the small-sample bias and the selection bias. For example, only hospitalized patients were included in our research. We also failed to allocate an independent cohort for external validation of our model, which would affect the reliability of our model to some extent. We expected that our model could be validated with an increase in the number of cases or the participation of multiple medical centers. Third, due to the limited follow-up of the study, the time course for survival analysis was short, only allowing for a description of the 1- and 2- year prognosis. The long-term prognosis requires longer follow-up. Furthermore, due to imperfect conditions, some patients did not complete appropriate imaging examinations at the third, sixth, and 24th month of follow-up, which affected our analysis of variables describing imaging characteristics and the exact efficacy of fistula healing.

In conclusion, we have developed a predictive model using the existence of complex fistula, disease activity at baseline and IFX efficacy at 6 months to predict the long-term outcome of luminal fistulizing CD patients. Due to the limited sample size, our prediction model cannot form an efficient nomogram for the time being. But with more cases prospectively included in our cohort, the model could be validated and present potential clinical utility with further validation and optimization.

## Data Availability

The raw data supporting the conclusion of this article will be made available by the correspondence author via email *liji0235@pumch.cn*, without undue reservation.

## Abbreviations

BMI: Body mass index
CD: Crohn’s disease
CDAI: Crohn’s Disease Activity Index
CI: Confidence interval
C-index: Concordance index
CRP: C-reactive protein
CT: Computerized tomography
ESR: Erythrocyte sedimentation rate
HR: Hazard ratio
IFX: Infliximab
lfCD: Luminal fistulizing Crohn’s disease
MR: Magnetic resonance
MRI: Magnetic resonance imaging
pfCD: Perianal fistulizing Crohn’s disease
TNF-α: Tumor necrosis factor-α
